# Does Bullying Occur behind Closed Doors? Agreement of Bullying Reports between Parents and Children and Its Differential Associations with Child Outcomes

**DOI:** 10.3390/children9101440

**Published:** 2022-09-22

**Authors:** Slava Dantchev, Martina Zemp

**Affiliations:** 1 Department of Clinical and Health Psychology, University of Vienna, 1010 Vienna, Austria; 2 Faculty of Psychology, Sigmund Freud University, 1020 Vienna, Austria

**Keywords:** bullying, parent-child, child outcomes, agreement, emotional problems, conduct problems, sibling, peer, APIM, family systems

## Abstract

The present study was aimed at examining the level of agreement between parent and child perceptions of sibling and peer bullying (victimization and perpetration), and investigating whether any differential associations with child emotional and conduct problems could be identified across raters. The actor-partner interdependence model (APIM) was utilized in order to statistically account for the non-independence of the parent-child dyad. The study was based on a sample of 142 parent-child dyads (children: *M*_age_ = 12.3 years; parents: *M*_age_ = 44.4 years) and employed an online survey design. Bullying experiences and child outcomes were assessed via parent- and self-report. Intraclass correlation analysis revealed a moderate level of agreement between parent- and child-reports of sibling and peer bullying victimization. Low to moderate levels of parent-child agreement emerged for sibling perpetration and low agreement for peer perpetration. Moreover, APIMs found that parent- and child-reports of bullying were differentially associated with child adjustment. The results of this study suggest that child- and parent data each add additional and unique information into the big picture. Thus, our findings argue for the utility of integrating parent and child perspectives simultaneously in research and clinical practice, in order to uncover the complex reality of child functioning in the context of the family system.

## 1. Introduction

Bullying is understood as frequent and repeated aggressive behavior that is intended to inflict harm and involves an element of power imbalance [1]. Bullying typically consists of direct (e.g., threatening, hitting, teasing or calling others nasty names) and/or indirect forms (e.g., being socially excluded, ignored or having others spread rumors about oneself) and has been documented to occur at home between siblings [2] and at school between peers [3]. Bullying experiences among children and adolescents are common, with mean prevalence estimates of 36% for victimization and 35% for perpetration in the peer context [4], and a range from 15–50% for victimization and 10–40% for perpetration in the sibling context [2]. In addition to those children who act as pure victims or pure bullies, some children have been found to be victims, who also fight back and bully others (i.e., bully/victims) [3]. Moreover, there is a bulk of empirical evidence showing a robust association between involvement in sibling and peer bullying during childhood and a range of negative mental health consequences lasting well into adulthood [5,6,7,8]. Recent work has found that bullying may be most harmful when experienced across multiple contexts (sibling and peer), thus investigating multiple bullying forms across contexts is essential [5,9].

### 1.1. Agreement of Bullying Reports between Children and Parents

Bullying in childhood and adolescence is most commonly assessed using self-report measures, the method of choice for prevalence estimates [10]. Self-reports have been argued to be particularly suitable, as there are many “covert” forms of bullying (e.g., spreading rumors or social exclusion) that are difficult to be reported by uninvolved individuals. Furthermore, bullying often occurs behind closed doors and, thus, parents or teachers may underestimate or be unaware of the problem behavior [2,10,11]. Parents have been found to be more aware of their children’s bullying experiences compared to teachers [12,13,14], and youth confide about their bullying experiences (as victims or perpetrators) to their parents more often as compared to their teachers [15]. 

It is crucial for parents to recognize whether their child is involved in bullying, as either a victim or a perpetrator, in order to respond appropriately. Parents can comfort their children by offering emotional support to their child’s distress, guide their children in the process of developing strategies to deal with their difficulties, and they can also take action themselves to intervene in and prevent future bullying [16,17,18]. Supportive family environments have been shown to be directly linked with help-seeking behavior of children exposed to bullying as a victim, and increased levels of parental response and communication about bullying for children engaging in perpetration [13]. In contrast, parents who are completely unaware of their children’s bullying involvement may be unable to support them accordingly. Thus, if children do not confide in their parents or if bullying is not a salient topic at home, it may remain hidden. 

One common way of assessing the extent to which parents are aware of their children’s bullying involvement is to explore the agreement across child- and parent-reports of bullying. Studies have robustly found low rates of parent-child agreement in the general population [13,19,20,21,22,23] as well as in clinical samples [24]. Agreement levels were higher for victimization as compared to perpetration, suggesting that children may be more likely to talk to their parents about their victimization experiences [13]. In one study, parents misjudged their victimized children’s readiness to seek out help and likewise held inaccurate beliefs about whether their children would engage in perpetrating bullying [19]. Similarly, parents have even been found to be largely unaware of sibling bullying at home [2]. On the one hand, bullying between siblings is frequently dismissed by parents as a normative part of growing up [25]. On the other hand, sibling bullying has been described to occur in the absence of parental supervision [26]. While young people have reported to most likely confide in their parents about bullying among siblings [27], there is no previous study, to our knowledge, that has explored to what extent children and parents agree in their reports about sibling bullying. 

Taken together, scholars agree that it is crucial to employ multiple perspectives and integrate a multi-informant approach when studying bullying [28]. Moreover, there is a clear and urgent need to sensitize parents and raise awareness about bullying, as well as equip parents with effective strategies to support their children when faced or made aware of bullying [16]. 

### 1.2. Agreement of Reporting Child Outcomes between Children and Parents 

In general, multi-informant approaches are considered best-practice for evaluating psychological problems in children and adolescents [29]. In a meta-analysis of 119 studies, a weak mean correlation was found between child- and parent-reports of overall emotional and behavioral problems in children and adolescents [30]. More recent work has confirmed these findings, similarly reporting low to moderate agreement across parent- and child-reports of child adjustment in both community [31,32,33,34,35,36] and clinical samples [37,38,39,40]. Interestingly, there is some research suggesting that parents may be better at reporting child externalizing problems, while children’s self-reports may reflect their own internalizing problems more accurately [33,38,39,40]. Methodologically, however, the literature suggests that different informants may contribute unique, complementary information that are both valid, in order to better understand children’s emotional and behavioral problems [36]. Moreover, low agreement between informants has been argued to reflect differing information that may vary by situation, as opposed to a lack of validity associated with either informant [29].

### 1.3. A Family Systems Approach 

**Figure 1:**
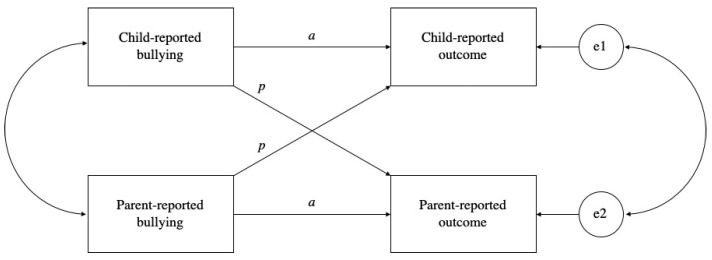
Conceptual depiction of the actor-partner interdependence model (APIM) of the impact of child bullying on child outcomes.

Family systems theory (FST; [41]) posits that family subsystems (e.g., parent-child, interparental, sibling relationships) should be understood as interdependent units, mutually influencing one another in their emotions, thoughts, and behavior. Thus, in order to better understand the intricate ways in which child behavior and well-being are embedded, it is deemed necessary to explore different family sub-systems or perspectives collectively. Along these lines, the actor-partner interdependence model (APIM; [42,43]) offers a statistical framework in order to model dyadic interpersonal relationships, thereby integrating the tenets of interdependence in two-person relationships [44,45]. Figure 1 presents a conceptual depiction of the APIM, illustrating both the actor and partner effects, two of the central components of the APIM, applied to the current study. The actor effects measure how much a person’s perception of an outcome (e.g., child-reported emotional problems) is affected by his or her own perception of a predictor (e.g., child-reported bullying experiences), as illustrated by the two paths labeled *a* in Figure 1. The partner effects, on the other hand, measure how much a person’s perception of an outcome (e.g., child-reported emotional problems) is affected by another person’s perception of a predictor (e.g., parent-reported bullying experiences of the child), as illustrated by the two paths labeled *p* in Figure 1. Employing the APIM thereby allows for a deeper understanding of the interdependence between child and parent perspectives on child bullying involvement and its outcomes. It can simultaneously investigate whether the rater’s perspective (child and/or parent) is a determining factor in explaining the relationship between child bullying and child outcomes, whilst controlling for the intrinsic non-independence within a dyadic (in this instance, parent-child) relationship.

### 1.4. Current Study 

The aims of the present study were to explore the agreement between parent- and child-reports of children’s sibling and peer bullying victimization and perpetration, and to examine differential associations with child outcomes in a sample of 142 parent-child dyads living in Austria. This study adds to the current literature by, for the first time, integrating parent and child perspectives of bullying victimization and perpetration across multiple contexts (sibling and peer), as well as child adjustment (emotional and conduct problems) using the APIM [44,45]. 

The potential usefulness of integrating child and parental perspectives of both bullying experiences and child outcomes has not been considered previously. Thus, the findings of the current study may inform future work in the utility of employing both parent and child perceptions of bullying and provide indications of their usefulness in predicting youth’s adjustment and well-being. In order to inform methodological decisions revolving around assessment in research as well as clinical practice, it is important to examine whether multiple reporters’ data should be considered in isolation (e.g., whether it suffices to rely on any one informant alone), in union (e.g., as multiple sources strengthening the validity of the underlying construct), or as complements (e.g., exploring both perspectives simultaneously but as separate constructs, each adding additional and unique information into the big picture). The current work can further inform the ongoing debate relating to cross-informant agreement [10]. Moreover, there are no previous studies on sibling bullying that have examined the agreement across parent- and child-reports. 

The current study will address the following research questions and hypotheses: (1) What is the level of agreement among parents and children in overall perceptions of sibling and peer bullying victimization and perpetration? We hypothesized that there would be a low to moderate level of agreement between parents and children concerning bullying victimization; the agreement between parent- and child-reports of bullying perpetration is expected to be weaker. (2) Are there actor effects (e.g., child-reported bullying is associated with child-reported outcomes) among parents and children in the link between bullying victimization and perpetration (sibling and peer context) and child emotional and conduct problems? We expected that parent and child perceptions of bullying (victimization and perpetration) would be positively associated with their own perception of child outcomes (emotional and conduct problems). Specifically, we hypothesized these actor effects to be strongest between victimization and emotional problems, and between perpetration and conduct problems. Actor effects for children were expected to be stronger than for parents, as predictor and outcome are related to the child’s behavior itself. (3) Are there partner effects (e.g., child-reported bullying is associated with parent-reported child outcomes) among parents and children in the link between victimization and perpetration (sibling and peer context) and child emotional and conduct problems? We expected to find partner effects from child-reported bullying (victimization and perpetration) to parent-reported child outcomes (emotional and conduct problems), with the strongest positive association between child-reported victimization and parent-reported conduct problems. We did not expect to find any partner effects from parent-reported bullying to child-reported outcomes. 

Given the lack of research in this area, it was difficult to speculate on the differential effects for sibling and peer bullying. For this reason, no specific hypotheses were formulated for the different contexts of bullying.

## 2. Materials and Methods

### 2.1. Participants

The sample of the current report included a total of 142 parent-child dyads. Children (53.5% male) were aged 9 to 15 years (*M* = 12.26, *SD* = 1.24), while parents (11.3% male) were aged 34 to 59 years (*M* = 44.39, *SD* = 1.89). The majority of families (97.9%) were Austrian. Fifty-three parents (37.3%) reported holding a university degree, while 88 (67.2%) reported earning more than the average household income (>3000 Euro, [46]). A total of 109 (77.9%) parents reported having more than one child. Out of these parents, 62.4% had two children, 25.5% had three children, and 10.1% had four or more children. All children having at least one sibling constituted the sibling subsample for all analyses concerning sibling bullying (see below). Only three (2.2%) children and seven (5.1%) parents reported being in current psychotherapeutic or psychiatric treatment. 

### 2.2. Procedure

The current study employed an online survey design using the software SoSci Survey [47]. Parents and children received separate study links in order to permit independent participation. To be included in this study, data from both a parent and a child from the same family (matched via alphanumerical codes) had to be available, thus only parent-child dyads who both completed the online survey (parent or child versions respectively) were included. Parent-child dyads were further required to speak German. Moreover, children had to (1) receive parental consent and (2) be aged 9 to 15 years in order be included. 

Participants were recruited from regional schools as well as via social media platforms for parents and families. Participating schools (*n* = 14) supported the recruitment process by sharing the online study link and corresponding study information with parents. Recruitment via the online route was conducted by making the relevant study material and study link available directly via the social media platforms. Two recruitment phases took place: (1) April 2020–June 2020; and (2) January 2021–April 2021. Participation was voluntary and could be withdrawn at any given time. Study links were shared exclusively with parents in order to ensure parental consent prior to allowing child participation. Child study links were made available to parents once they had read through all of the relevant study information and agreed to share the corresponding link with their children. Parents were given the choice of sharing the link with their children and/or participating themselves. Ethical approval for the study was obtained from the Institutional Review Board of the University of Vienna (Reference Nr.: 00480; Date of approval: 19 November 2019).

### 2.3. Measures

#### 2.3.1. Sibling and Peer Bullying

Sibling bullying victimization and perpetration was assessed via two adapted items from the Revised Olweus Bullying Questionnaire [1]. Youth were provided with a definition of sibling bullying and asked to report the frequency of experiencing sibling bullying (victimization) and the extent to which they had ever bullied a sibling (perpetration) in the last six months. Parents were given the same definition and similarly asked to report the frequency of their children’s sibling bullying involvement (victimization and perpetration) in the past six months. Responses were given on a 4-point Likert-scale (1 = *never*; 2 = *seldom: 1–3 times during past six months*; 3 = *frequently: more than three times during past six months*; 4 = *very frequently: at least once per week*) and were treated as continuous variables with higher scores reflecting higher levels of victimization (child-reports: *M* = 1.60, *SD* = 0.95; parent-reports: *M* = 1.93, *SD* = 1.07) or perpetration (child-reports: *M* = 1.50, *SD* = 0.85; parent-reports: *M* = 1.86, *SD* = 1.01), respectively. 

Peer bullying victimization and perpetration were assessed via a German-language bullying screening [48,49]. The screening was adapted from a previously validated bullying questionnaire [50], which was developed using large national samples across the UK and Germany. The screening includes four items that address: (1) direct peer victimization (e.g., being threatened, hit, called nasty names or teased); (2) direct peer perpetration (e.g., threatening, hitting, teasing or calling others nasty names); (3) indirect peer victimization (e.g., being socially excluded, ignored or having others spread rumors about oneself); and (4) indirect peer perpetration (e.g., socially excluding, ignoring or spreading rumors about others). A definition with examples was provided for each bullying form. 

Children were then asked to report the frequency of experiencing peer bullying (victimization) and the extent to which they had ever bullied a peer (perpetration) in the last six months. Parents were likewise asked to report the frequency of their children’s peer bullying involvement (victimization and perpetration) in the last six months. Responses were given on the same 4-point Likert-scale as for sibling bullying above. Likert-scale variables were treated as continuous variables. For peer victimization and perpetration, the highest frequency of either item (direct and indirect) was used to assign children their peer victimization and perpetration score respectively (e.g., if a child reported experiencing direct victimization frequent, but only seldom indirect victimization, a score of 3 = frequently was assigned; [5]). Higher scores reflected greater victimization (child-reports: *M* = 1.38, *SD* = 0.64; parent-reports: *M* = 1.53, *SD* = 0.67) or perpetration (child-reports: *M* = 1.10, *SD* = 0.25; parent-reports: *M* = 1.17, *SD* = 0.32), respectively.

#### 2.3.2. Emotional Problems

Emotional problems were assessed via self- and parent-reports of the respective subscale of the Strengths and Difficulties Questionnaire (SDQ; [51]), adapted for use in German [52]. Children were asked to report on five items assessing emotional problems over the past six months (e.g., “I get a lot of headaches, stomach-aches or sickness”). Parents were asked to report on the analogous five items (e.g., “Often complains of headaches, stomach-aches or sickness”). Responses were given on a 3-point Likert-scale (1 = *not true*, 2 = *somewhat true*, and 3 = *certainly true*). A mean score was computed, resulting in a possible range from 1 to 3, with higher scores reflecting higher levels of emotional problems (child-reports: *M =* 1.45, *SD* = 0.47; parent-reports: *M* = 1.37, *SD* = 0.45). Cronbach’s alpha was 0.77 for children and 0.82 for parents in the current sample.

#### 2.3.3. Conduct Problems

Conduct problems were assessed via the corresponding subscale of the Strengths and Difficulties Questionnaire (SDQ; [51,52]) using youth self-reports and parent-reports. Child-reported conduct problems included five items (e.g., “I get very angry and often lose my temper”). Parents were asked to report on the analogous five items (e.g., “Often loses temper”). Responses were given on a 3-point Likert-scale (1 = *not true*, 2 = *somewhat true*, and 3 = *certainly true*). A mean score was computed in order to reflect conduct problems, resulting in a possible range from 1 to 3, with higher scores representing greater conduct problems (child-reports: *M* = 1.19, *SD* = 0.27; parent-reports: *M* = 1.27, *SD* = 0.28). In the child-report subscale the second item was not included in order to increase the internal consistency of the scale. Cronbach’s alpha was 0.53 for children and 0.51 for parents in the current sample.

#### 2.3.4. Control Variables 

Youth gender (0 = female; 1 = male), age (in years), and COVID-19 recruitment phase (0 = April 2020 through June 2020; 1 = January 2021 through April 2021) were included as control variables across all statistical models. It was important to account for schooling irregularities and/or pandemic-related influences as the data was collected during the COVID-19 pandemic. Schools in Austria were closed in mid-March 2020 and online distance-learning measures were imposed. Hybrid measures were introduced in mid-May 2020 allowing for a combination of face-to-face and online learning opportunities. Children were permitted back to school starting September 2020, however, another round of national school closures were announced in November 2020. Starting in February 2021 a hybrid solution was re-introduced. 

#### 2.3.5. Statistical Analysis

IBM SPSS Statistics [53] was utilized for all preliminary and descriptive analyses, as well as the examination of child and parent agreement levels, while Mplus 8.1. [54] was used for all structural equation modeling (SEM). First, descriptive statistics were calculated for all key study variables. Second, in order to assess the level of agreement across parent- and child-reports of bullying, intraclass correlation coefficients (ICCs) were calculated separately for bullying victimization and perpetration across context (sibling and peer; research question 1). Third, in order to explore whether childhood bullying experiences were differentially associated with child outcomes depending on the perspective (parent- vs. child-reports), APIMs were computed in Mplus (research questions 2 and 3). Separate APIMs were calculated for victimization and perpetration. The APIM allows to measure interdependence within dyadic relationships by capturing actor and partner effects [44,45]. Control variables were regressed onto the dependent variables. A post-hoc power analysis was performed using the online tool APIMPower [55], once the APIMs were computed and parameter estimates were derived. A number of model fit indices were used to evaluate our models: (1) root-mean-square error of approximation (RMSEA), (2) comparative fit index (CFI), (3) Tucker–Lewis Index (TLI), and (4) standardized root-mean-square residual (SRMR). Indicators for a good model fit are as follows: >0.95 for CFI and TLI, <0.06 for RMSEA and <0.08 for SRMR [56]. 

## 3. Results

Descriptive statistics across the main study variables for the total sample are shown in Table 1. Separate intercorrelation matrices of the key study variables across the sibling subsample (i.e., all children having at least one sibling; *n* = 108; see Table 2) and the peer sample (i.e., full sample; *n* = 142; see Table 3) were computed. Sibling victimization and perpetration based on the same reporter (child-reports: *r* = 0.82; parent-reports: *r* = 0.82) were found to correlate the highest, suggesting strong overlap across roles (i.e., children reporting more victimization also reported more perpetration themselves). Peer victimization and perpetration were moderately correlated across reporters. Child outcomes (emotional and conduct problems) were moderately correlated within and across reporters for both sibling and peer bullying. Surprisingly, no correlations across sibling bullying and child outcomes were found, irrespective of reporters. Peer bullying and child outcomes on the other hand were correlated across reporters, with the exception of child-reported peer perpetration and parent-reported child outcomes as well as parent-reported peer perpetration and emotional problems. 

### 3.1. Agreement between Parent- and Child-Reports of Bullying

Two-way mixed-effect intraclass correlation analyses (see Table 4) revealed that the inter-rater reliability between child- and parent-reports of bullying victimization (sibling and peer) was moderate, with peer victimization showing the highest level of agreement (ICC = 0.58; 95% CI [0.45–0.68]) across all bullying forms. The agreement of sibling perpetration was between low to moderate, while the agreement of peer perpetration was low. Emotional problems showed moderate agreement, while conduct problems showed low agreement, across both the sibling subsample and peer sample. 

### 3.2. Agreement between Parent- and Child-Reports of Bullying

#### 3.2.1. Actor Effects in the Link between Bullying and Child Outcomes

Actor effects for sibling bullying and peer bullying can be found in Table 5 and Table 6, respectively. Actor effects were identified for parent perceptions of sibling victimization and parent-report of child conduct problems (model 2: *β* = 0.107, *p* = 0.001). Actor effects were also found for parent perceptions of sibling perpetration and parent-report of child conduct problems (model 4: *β* = 0.172, *p* < 0.001), with greater bullying being linked to more conduct problems from parental view. Actor effects for children were found between peer victimization and conduct problems (model 6: *β* = 0.143, *p* = 0.012). With regard to peer victimization and emotional problems, actor effects emerged for both child-reports (model 5: *β* = 0.180, *p* = 0.003) and parent-reports (model 5: *β* = 0.197, *p* = 0.003). Moreover, actor effects for children also surfaced for peer perpetration and emotional problems (model 7: β = 0.252, *p* = 0.018); thus, children who reported more bullying against peers also reported higher levels of emotional problems themselves. Finally, actor effects emerged for parent-reported peer perpetration and parent-report of conduct problems (model 8: *β* = 0.166, *p* = 0.002). The post-hoc power analysis revealed that power across all actor effects ranged from 0.051 to 1.00. A detailed overview of all parameters associated with the power calculations across models can be found in the online supplementary material (see Supplementary Table S1 for sibling bullying and Supplementary Table S2 for peer bullying). All models fit the data well, as indicated by the model fit indices in the respective table.

#### 3.2.2. Partner Effects in the Link between Bullying and Child Outcomes

Partner effects for sibling bullying and peer bullying can be found in Table 5 and Table 6, respectively. There were no partner effects across parent- and child-reports of bullying victimization and child outcomes. A partner effect was identified between child-reported sibling perpetration and parent-reported emotional problems (model 3: *β* = −0.107, *p* = 0.032), indicating that increased levels of child-perceived sibling perpetration was linked to lower levels of child emotional problems as observed by parents. The post-hoc calculated power across all partner effects ranged from 0.050 to 0.473. A detailed overview of all parameters associated with the power calculations across all models can be found in the online supplementary material (see Supplementary Table S1 for sibling bullying and Supplementary Table S2 for peer bullying). All models fit the data well, as indicated by the model fit indices in the respective table.

#### 3.2.3. Control Variables

Child age, child gender, and COVID-19 recruitment phase were included as control variables across all APIM models. Each confounder was regressed onto the outcome variables. The COVID-19 phase was found to be significant confounder models 2, 4, 6, and 8, such that children recruited in phase 1 (recruitment occurred between April 2020 and June 2020) were found to have higher levels of conduct problems. Gender further emerged as a significant confounder in models 5 and 7, suggesting that girls were found to have more emotional problems. 

## 4. Discussion

The present study was aimed at examining the level of agreement between parent and child perceptions of sibling and peer bullying and investigating whether any differential associations with child emotional and conduct problems could be identified across raters, taking into account statistically the non-independence of the parent-child dyad. The potential usefulness of integrating parent- and child-reports of both bullying experiences and child outcomes has not been considered previously. Adopting a family systems framework [41], this study was the first thus far to integrate parent and child perspectives of child adjustment (emotional and conduct problems) as well as bullying (victimization and perpetration) across multiple contexts (sibling and peer) using the APIM [44,45]. 

There is a bulk of empirical evidence identifying sibling and peer bullying in childhood and adolescence as a significant risk factor compromising well-being and predicting a range of mental health and behavioral outcomes in early- to late adulthood [5,6,7,8]. Early parental recognition and support in the face of bullying has been discussed as a central factor towards intervening effectively against bullying [57,58] and reducing negative developmental trajectories in youth [5,59]. Thus, it is crucial for parents to be aware of their children’s involvement in bullying. As expected, results from our intraclass correlation analysis found a moderate level of agreement between parent- and child-reports of sibling and peer bullying victimization. On the other hand, low to moderate levels of parent-child agreement emerged for sibling perpetration and low agreement for peer perpetration. Thus, in line with our hypothesis the agreement between parent- and child-reports of bullying perpetration was found to be weaker as compared to bullying victimization. It appears that children are likely more prone to disclose or be open about their victimization experiences vis-à-vis their parents than about bullying perpetration, mirroring results in other studies [13]. Moreover, our findings largely resonate with previous studies in the field that have robustly reported low levels of agreement between parent- and child-reports of peer bullying [13,19,20,21,22,23]. As such, our results support the notion that scholars should employ a multi-informant approach in future studies on bullying [28]. It is important to note that the findings of the current study were based on the perspective of one parent only. It is possible that parents have different degrees of knowledge or awareness of their children’s bullying experience, thus future work should aim to incorporate both parental views. Similarly, teachers assume a predominant supervisory role in school, therefore it may be useful for scholars to additionally ask teachers about their perceptions of peer bullying as well. Moreover, our findings add novel insights to the current literature. With no previous work on inter-rater agreement within the sibling context, we recognize the importance of integrating both parent- and child-reports of sibling bullying in future work, as both raters may carry unique sources of information, similar to the peer context. 

Next, we turn to actor effects of bullying and child outcomes. We hypothesized that parent- and child-reports would be positively associated with their own perceptions of child outcomes. Only about half of all actor effects emerged as significant, thereby confirming this hypothesis in part only. Firstly, positive actor effects were found for parent perceptions of sibling victimization and perpetration and their own perception of child conduct problems, such that when parents perceived their child as being more often involved as a victim or perpetrator of sibling bullying, they also noticed higher levels of conduct problems in their child. Second, positive actor effects for children were found between peer victimization and conduct problems. Hence the more children disclosed being victimized by peers, the more conduct problems they reported. With regards to peer victimization and emotional problems, positive actor effects emerged for both child- and parent-reports. In other words, the more children were being victimized, either reported by the children themselves or their parents, the greater was the level of emotional problems in the children’s and parents’ perspectives. Similarly, a positive actor effect for child-reported peer perpetration and emotional problems was found, with those children who disclosed more peer bullying also reporting more emotional problems. Lastly, positive actor effects were also identified for parent-reported peer perpetration and conduct problems, so children whose parents perceived them as peer bullies were also seen as displaying more externalizing behavior. These findings generally map well onto previous work indicating robust links between bullying (victimization and perpetration) with internalizing and externalizing problems [9,60,61]. It is, however, difficult to make direct comparisons, as there is no work to date which has compared differential links between bullying across contexts and child outcomes in the same dyadic model. 

We had expected that associations would be strongest between victimization and emotional problems as well as between perpetration and conduct problems. Our findings were mixed and inconclusive in this respect. In a similar way, the hypothesis that actor effects for children would be stronger than for parents was discounted by our findings. From a purely quantitative point of view, there were more significant parent actor effects than child actor effects. Additionally, it is interesting that there were only two parent actor effects across all models of sibling bullying and no significant paths when considering the children’s perspective. How could these discrepancies as well as the lack of consistent actor effects across different models be explained? One possible avenue to explore might be methodological caveats of the current study. The comparatively low proportion of child actor effects may, for instance, be a result of our sampling and design method. First, the online study design employed may have reduced the validity of our data. Parents who chose to participate were responsible for providing their children with the online survey link. It is therefore possible that children completed the questionnaires in the presence of their parents, leaving child-reports particularly susceptible to response bias [62]. Children may thus have been less likely to respond truthfully and fully disclose their own experiences of bullying and outcomes. Moreover, parents who themselves decided to partake in the present study and encouraged their children to do so may also represent a particularly involved family population who, by baseline, is more attuned to their children’s lives, however this remains purely speculative. 

Moving onto partner effects, it was surprising to find only one single partner effect: That is, our results revealed a negative association between child-reported sibling perpetration and parent-reported emotional problems, indicating that increased levels of sibling perpetration perceived by the child was related to lower levels of child emotional problems as rated by parents. This is an interesting finding, which for one illustrates the way in which partly masked links in families can be uncovered through the APIM. At the same time however, it also represents a counterintuitive finding at first glance. Evolutionary perspectives on sibling aggression can lend some support in enriching our understanding of these results. Evolutionary theories posit that sibling aggression is driven by competition over resources (i.e., parental attention; [63]). Indeed, there is robust empirical evidence suggesting that sibling bullying is used as an evolutionarily driven strategy to help secure social dominance within the family [64,65]. Along these tenets we may speculate that if indeed sibling bullying perpetration is an evolutionarily adaptive strategy, children who bully their siblings should fare better, a premise which is reflected in our findings: children reporting perpetrating sibling bullying were found to display lower levels of emotional problems as perceived by their parents. However, partner effects were not found between parent-reported sibling perpetration and emotional problems. It may be that successfully securing coveted family resources is reserved for those children who are more skilled at bullying their siblings behind closed doors, leaving the bullying undetected by parents, but these assumptions remain strictly speculative and remit further exploration in future studies. 

We had expected partner effects between child-reported bullying (victimization and perpetration) and parent-reported child outcomes (emotional and conduct problems), with the strongest association between child-reported victimization and parent-reported conduct problems. Hence, we could not confirm this assumption with our data. The absence of partner effects from parent-reported bullying to child-reported outcomes, however, was in line with our hypothesis. In light of the lacking partner effects, one may speculate about the utility in employing a dyadic (parent-child) approach when assessing the associations between bullying and child outcomes. That said, we would strongly doubt that the consideration of only one perspective (i.e., the child or one parent) is the more suitable approach in this field. However, it might be premature to draw such conclusions. Rather, it remains necessary to consider these in the view of past work. Past studies have reported on low to moderate rates of parent-child agreement of bullying [2,13,19,20,21,23] and child outcomes [31,32,33,34,35,36]. Considering the low to moderate levels of parent-child agreement in reports observed in our own data, it might be that, in line with methodological debates in the field, both children and parents contribute unique, complementary information that are both valid [29], and thus they should both be examined simultaneously. 

### 4.1. Practical Implications

The findings of the current study suggest the utility of using child- and parent data as complements in exploring both perspectives simultaneously, each adding additional and unique information into the big picture. Hence, our findings hold promise in informing future work as well as methodological decisions revolving around assessment in research and clinical practice. Taken together, although parent-child reports of bullying and child outcomes did not correlate highly, they were also not independent of each other, thus our findings argue for the integration of both perspectives as invaluable sources of information about child behavior and well-being. These findings resonate with recent transitions in the field which place an increasingly strong emphasis on the importance of integrating a family systems approach within clinical child and adolescent psychology [66,67]. Families are regarded as an organized whole and elements within this system are mutually interdependent, hence behaviors, beliefs and emotions of family members are inextricably interconnected [41]. Along these lines, our findings argue that parent and child perspectives should be put more firmly on the agenda of practitioners by routinely integrating both of these in the context of the diagnostic process of assessing bullying and child adjustment in clinical practice. As such, the present findings may be particularly helpful in guiding pediatric and primary care providers in determining the appropriate methods to screen for bullying, given the current lack of systematic screening protocols in the area [68]. Placing a greater emphasis on multiple family informants in both research and practice holds great promise in elucidating the intricate interdependency in this domain. Exploring single associations separately may lead to statistical artefacts, as it is not possible to statistically control for the variance of other family members. Thus, it remains essential for future studies to continue integrating parent and child perspectives simultaneously within the same statistical models in order to uncover the complex reality of how child functioning is embedded within family systems. Last, in line with a contemporary biopsychosocial perspective on psychopathology, the complex interplay between risk and resilience should be considered. The family system can act as both a primary source of risk, as well as an essential source of protective factors. Future work may therefore benefit from additionally examining family factors associated with resilience in the context of bullying and child adjustment. 

### 4.2. Limitations 

A few limitations merit further discussion. First, the sample size was relatively small and not representative of the general population; therefore, generalizability of the present results is limited. Second, a cross-sectional design was employed that prohibits us from drawing causal conclusions regarding the reported associations. Replication studies with larger samples utilizing a longitudinal design (i.e., measuring bullying and child outcomes over time) are needed. Third, our post-hoc power analyses revealed a wide variation of power estimates across models and actor-partner effects; thus findings should be interpreted with caution. Fourth, we drew from questionnaire data for all constructs. Although we had reports from parents and children, studies using observational assessment are important to replicate the findings and to evaluate the methodological robustness of our results. Similarly, integrating focus groups and interview approaches can help shed light on how parents, as well as teachers, view children’s involvement in bullying. Fifth, we used brief screening measures to assess bullying and these may not have been sensitive enough to capture bullying in a differentiated manner. Sixth, our conduct problems measure was found to have a poor internal consistency. The low reliability scores are in correspondence with previous studies using the German SDQ [69,70] and might be explained by the multidimensional nature of this screening tool. Respective findings should be treated with reservation. Last, our online study design may have compromised the validity of our data, given that participants completed all questionnaires in the absence of a controlled environment. 

## 5. Conclusions

Utilizing a family systems framework embedded within the APIM has provided a valuable avenue in providing us with initial insights into the interdependence between child and parent perspectives on child bullying involvement and its outcomes. The simultaneous investigation of child and parent perceptions was a determining factor in coming one step closer towards better understanding the associations between children’s bullying experiences and their emotional and conduct problems, whilst controlling for the intrinsic interdependency within this natural dyadic (in this instance, parent-child) relationship. Future replication work in this domain is urgently needed in order to further elucidate the intricate dynamics of employing a multi-rater approach in assessing child adjustment within families.

## Figures and Tables

**Table 1:** Descriptive statistics of key study variables.

	Child-Report	Parent-Report
Variables	*M* (*SD*) or *n* (%)	*M* (*SD*) or *n* (%)
Sibling subsample (*n* = 108)		
Sibling victimization	1.60 (0.96)	1.97 (1.07)
Sibling perpetration	1.51 (0.85)	1.90 (1.00)
Emotional problems	1.45 (0.47)	1.37 (0.44)
Conduct problems	1.27 (0.37)	1.29 (0.28)
Child age	12.22 (1.20)	-
Male gender	58 (53.7)	-
COVID-19 phase 1	49 (45.4)	-
Peer sample (*n* = 142)		
Peer victimization	1.56 (0.86)	1.72 (0.81)
Peer perpetration	1.17 (0.41)	1.26 (0.46)
Emotional problems	1.45 (0.47)	1.38 (0.45)
Conduct problems	1.26 (0.36)	1.28 (0.28)
Child age	12.26 (1.24)	-
Male gender	76 (52.4)	-
COVID-19 phase 1	60 (41.4)	-

Note. Child age, child gender, and COVID-19 phase were included as control variable reported by study children only. COVID-19 phase 1 = recruitment occurred between April 2020 and June 2020.

**Table 2:** Intercorrelations of key study variables across the sibling subsample (*n* = 108).

Variables	1	2	3	4	5	6	7	8
Child-report								
1. Sibling victimization	-							
2. Sibling perpetration	0.82 **	-						
3. Emotional problems	0.36	0.10	-					
4. Conduct problems	0.11	0.14	0.28 **	-				
Parent-report								
5. Sibling victimization	0.51 **	0.36 **	0.05	0.15	-			
6. Sibling perpetration	0.49 **	0.51 **	0.05	0.25 *	0.82 **	-		
7. Emotional problems	−0.01	−0.15	0.52 **	0.05	0.14	−0.19	-	
8. Conduct problems	0.30 **	0.26 **	0.01	0.41 **	0.45 **	0.59 **	0.04	-

Note: * *p* < 0.05, ** *p* < 0.01.

**Table 3:** Intercorrelations of key study variables across the peer sample (*n* = 142).

Study Variables	1	2	3	4	5	6	7	8
Child-report								
1. Peer victimization	-							
2. Peer perpetration	0.37 **	-						
3. Emotional problems	0.34 **	0.19 *	-					
4. Conduct problems	0.37 **	0.22 *	0.34 **	-				
Parent-report								
5. Peer victimization	0.58 **	0.04	0.21 *	0.24 **	-			
6. Peer perpetration	0.06	0.24 **	−0.07	0.03	0.25 **	-		
7. Emotional problems	0.25 **	−0.07	0.59 **	0.16	0.38 **	−0.06	-	
8. Conduct problems	0.19 *	0.13	0.06	0.44 *	0.21 *	0.28 **	0.07	-

Note: * *p* < 0.05, ** *p* < 0.01.

**Table 4:** Parent-child agreement across bullying and child adjustment.

Variables			95% CI	
	*n*	ICC	Lower Limit	Upper Limit
Sibling subsample				
Sibling victimization	97	0.51	0.34	0.64
Sibling perpetration	96	0.50	0.33	0.78
Emotional problems	97	0.52	0.36	0.65
Conduct problems	97	0.40	0.21	0.55
Peer sample				
Peer victimization	129	0.58	0.45	0.68
Peer perpetration	126	0.24	0.06	0.39
Emotional problems	125	0.59	0.47	0.70
Conduct problems	124	0.43	0.27	0.56

Note: ICC = Intraclass correlation coefficient, 95 CI% = 95% confidence interval. Statistical analysis was conducted in SPSS; thus, it was not possible to apply full information maximum likelihood in order to account for missing data. Therefore, the N varies across variables and may not correspond to other indications of N throughout other tables. ICC < 0.50, poor; ICC between 0.50 and 0.75, fair; ICC between 0.75 and 0.90, good; ICC > 0.90, excellent.

**Table 5:** Actor-partner interdependence models of child- and parent-reports of sibling bullying and child outcomes (*n* = 108).

		95% CI			
	*b*	Lower Limit	Upper Limit	SE	*p*
Model 1					
Actor effects					
Child SV → Child EP	0.012	−0.069	0.100	0.051	0.818
Parent SV → Parent EP	0.075	0.001	0.153	0.045	0.093
Partner effects					
Child SV → Parent EP	−0.054	−0.129	0.048	0.054	0.318
Parent SV → Child EP	0.011	−0.075	0.086	0.047	0.819
Model Fit-Indices	χ^2^(6) = 6.692; RMSEA = 0.033 (0.000, 0.132); CFI = 0.981; TLI = 0.935; SRMR = 0.040				
Model 2					
Actor effects					
Child SV → Child CP	0.022	−0.055	0.114	0.050	0.663
Parent SV → Parent CP	0.107 **	0.054	0.164	0.033	0.001
Partner effects					
Child SV → Parent CP	0.026	−0.037	0.095	0.042	0.541
Parent SV → Child CP	0.037	−0.036	0.107	0.044	0.400
Model Fit-Indices	χ^2^(6) = 10.262; RMSEA = 0.081 (0.000, 0.163); CFI = 0.916; TLI = 0.719; SRMR				
Model 3					
Actor effects					
Child SP → Child EP	−0.007	−0.096	0.093	0.056	0.906
Parent SP → Parent EP	0.030	−0.045	0.096	0.043	0.492
Partner effects					
Child SP → Parent EP	−0.107 *	−0.186	−0.020	0.050	0.032
Parent SP → Child EP	0.021	−0.066	0.102	0.049	0.668
Model Fit-Indices	χ^2^(6) = 7.324; RMSEA = 0.045 (0.000, 0.139); CFI = 0.964; TLI = 0.881; SRMR = 0.041				
Model 4					
Actor effects					
Child SP → Child CP	0.012	−0.077	0.125	0.059	0.833
Parent SP → Parent CP	0.172 **	0.121	0.221	0.030	<0.001
Partner effects					
Child SP → Parent CP	−0.019	−0.087	0.053	0.043	0.663
Parent SP → Child CP	0.079	−0.002	0.151	0.046	0.087
Model Fit-Indices	χ^2^(6) = 10.948; RMSEA = 0.087 (0.000, 0.168); CFI = 0.928; TLI = 0.761; SRMR = 0.048				

Note. Reported coefficients reflect the unstandardized model results. SV = sibling bullying victimization, SP = sibling bullying perpetration, PV = peer bullying victimization, PP = peer bullying perpetration, EP = emotional problems, CP = conduct problems, Child = child-report, Parent = parent report. Control variables included: child age, child gender, and COVID-19 phase. Each confounder was regressed onto the outcome variables. Significant covariates: More conduct problems in recruitment phase 1 (models 2 and 4). * *p* < 0.05, ** *p* < 0.01.

**Table 6:** Actor-partner interdependence models of child- and parent-reports of peer bullying and child outcomes (*n* = 145).

		95% CI			
	*b*	Lower Limit	Upper Limit	SE	*p*
Model 5					
Actor effects					
Child PV → Child EP	0.180 **	0.073	0.270	0.061	0.003
Parent PV → Parent EP	0.197 **	0.099	0.312	0.066	0.003
Partner effects					
Child PV → Parent EP	0.017	−0.079	0.108	0.057	0.762
Parent PV → Child EP	0.006	−0.096	0.118	0.063	0.925
Model Fit-Indices	χ^2^(6) = 8.226; RMSEA = 0.051 (0.000, 0.127); CFI = 0.977; TLI = 0.923; SRMR = 0.039				
Model 6					
Actor effects					
Child PV → Child CP	0.143 *	0.047	0.223	0.057	0.012
Parent PV → Parent CP	0.049	−0.018	0.117	0.042	0.242
Partner effects					
Child PV → Parent CP	0.037	−0.017	0.101	0.036	0.298
Parent PV → Child CP	0.022	0.061	0.114	0.052	0.666
Model Fit-Indices	χ^2^(6) = 6.429; RMSEA = 0.022 (0.000, 0.112); CFI = 0.992; TLI = 0.974; SRMR = 0.040				
Model 7					
Actor effects					
Child PP → Child EP	0.252 *	0.073	0.427	0.106	0.018
Parent PP → Parent EP	−0.045	−0.165	0.102	0.082	0.585
Partner effects					
Child PP → Parent EP	−0.086	−0.223	0.056	0.085	0.307
Parent PP → Child EP	−0.118	−0.239	0.034	0.081	0.148
Model Fit-Indices	χ^2^(6) = 2.530; RMSEA = 0.000 (0.000, 0.056); CFI = 1.000; TLI = 1.161; SRMR = 0.023				
Model 8					
Actor effects					
Child PP → Child CP	0.186	0.030	0.353	0.096	0.054
Parent PP → Parent CP	0.166 **	0.069	0.250	0.054	0.002
Partner effects					
Child PP → Parent CP	0.053	−0.042	0.164	0.062	0.398
Parent PP → Child CP	0.003	−0.118	0.122	0.074	0.971
Model Fit-Indices	χ^2^(6) = 1.525; RMSEA = 0.000 (0.000, 0.000); CFI = 1.00; TLI = 1.329; SRMR = 0.018				

Note. Reported coefficients reflect the unstandardized model results. SV = sibling bullying victimization, SP = sibling bullying perpetration, PV = peer bullying victimization, PP = peer bullying perpetration, EP = emotional problems, CP = conduct problems, Child = child-report, Parent = parent report. Control variables included: child age, child gender, and COVID-19 phase. Each confounder was regressed onto the outcome variables. Significant covariates: More conduct problems in recruitment phase 1 (models 6 and 8); more emotional problems in girls (models 5 and 7). * *p* < 0.05, ** *p* < 0.01.

## Data Availability

The datasets presented in this study can be found in online repositories. The names of the repository/repositories and accession number(s) can be found below: [71]. The data presented in this study are openly available in OSF at DOI 10.17605/OSF.IO/8PSRJ, [71].

## Supplementary Materials

The following supporting information can be downloaded at: https://www.mdpi.com/article/10.3390/children9101440/s1, Table S1: Post-hoc power analysis of actor- partner interdependence models of child- and parent-reports of sibling bullying and child outcomes (*n* = 108); Table S2: Post-hoc power analysis of actor- partner interdependence models of child- and parent-reports of peer bullying and child outcomes (*n* = 145).
