# Supplementary material for: Does Bullying Occur behind Closed Doors? Agreement of Bullying Reports between Parents and Children and Its Differential Associations with Child Outcomes
Source: Children (Basel). 2022 Sep 22;9(10):1440. doi: 10.3390/children9101440 (PMC9600041; doi:10.3390/children9101440)
Supplement: Supplementary file 1 [file children-09-01440-s001.zip › children-1899469-supplementary.pdf]

Table S1. Post-hoc power analysis of actor- partner interdependence models of child- and parent-reports of sibling bullying and child outcomes (n = 108)

	Effect	Power	*n*	*df*	Beta	*r*	Partial *r*	ncp
**Model 1**								
Actor effect for child	0.024	0.055	108	105	0.024	0.037	0.021	0.210
Actor effect for parent	0.185	0.362	108	105	0.183	0.122	0.156	1.622
Partner effect for child	0.025	0.055	108	105	0.025	0.037	0.021	0.219
Partner effect for parent	-0.119	0.179	108	105	-0.188	-0.023	-0.101	-1.043
**Model 2**								
Actor effect for child	0.056	0.078	108	105	0.056	0.111	0.048	0.496
Actor effect for parent	0.403	0.943	108	105	0.370	0.412	0.329	3.570
Partner effect for child	0.107	0.156	108	105	0.107	0.136	0.092	0.948
Partner effect for parent	0.087	0.119	108	105	0.080	0.272	0.075	0.771
**Model 3**								
Actor effect for child	-0.012	0.051	108	105	-0.012	0.012	-0.010	-0.105
Actor effect for parent	0.069	0.092	108	105	0.068	-0.040	0.059	0.602
Partner effect for child	0.046	0.068	108	105	0.046	0.040	0.039	0.402
Partner effect for parent	-0.209	0.440	108	105	-0.206	-0.170	-0.175	-1.824
**Model 4**								
Actor effect for child	0.028	0.057	108	105	0.028	0.138	0.025	0.252
Actor effect for parent	0.610	10.00	108	105	0.212	0.227	0.183	1.908
Partner effect for child	0.212	0.473	108	105	0.212	0.227	0.183	1.908
Partner effect for parent	-0.056	0.079	108	105	-0.049	0.229	-0.049	-0.504

Note. Effect: the effect size value that was pre-specified based on the APIM results; Power: the level of power available to detect the effect; *n*: the number of complete dyads; *df*: the degrees of freedom associated with the number of dyads; Beta: the size of the effect in terms of the standardized regression coefficient; *r*: the zero-order correlation coefficient for the effect; partial *r*: the size of the effect in terms of the partial correlation coefficient; ncp: the non-centrality parameter for the effect.

Table S2. Post-hoc power analysis of actor- partner interdependence models of child- and parent-reports of peer bullying and child outcomes (n = 145)

	Effect	Power	*n*	*df*	Beta	*r*	Partial *r*	ncp
**Model 5**								
Actor effect for child	0.331	0.927	145	142	0.331	0.337	0.277	3.439
Actor effect for parent	0.356	0.957	145	142	0.351	0.370	0.296	3.699
Partner effect for child	0.010	0.051	145	142	0.010	0.199	0.009	0.104
Partner effect for parent	0.033	0.063	145	142	0.033	0.233	0.029	0.343
**Model 6**								
Actor effect for child	0.342	0.948	145	142	0.342	0.370	0.290	3.612
Actor effect for parent	0.142	0.319	145	142	0.149	0.218	0.125	1.500
Partner effect for child	0.050	0.082	145	142	0.050	0.245	0.044	0.528
Partner effect for parent	0.116	0.229	145	142	0.121	0.206	0.102	1.225
**Model 7**								
Actor effect for child	0.222	0.745	145	142	0.222	0.195	0.216	2.638
Actor effect for parent	-0.046	0.083	145	142	-0.046	-0.065	-0.045	-0.535
Partner effect for child	-0.116	0.278	145	142	-0.116	-0.063	-0.115	-1.378
Partner effect for parent	-0.079	0.150	145	142	-0.079	-0.090	-0.077	-0.919
**Model 8**								
Actor effect for child	0.212	0.703	145	142	0.212	0.213	0.206	2.510
Actor effect for parent	0.273	0.908	145	142	0.273	0.292	0.268	3.312
Partner effect for child	0.003	0.050	145	142	0.003	0.054	0.003	0.036
Partner effect for parent	0.078	0.156	145	142	0.078	0.144	0.079	0.946

Note. Note. Effect: the effect size value that was pre-specified based on the APIM results; Power: the level of power available to detect the effect; *n*: the number of complete dyads; *df*: the degrees of freedom associated with the number of dyads; Beta: the size of the effect in terms of the standardized regression coefficient; *r*: the zero-order correlation coefficient for the effect; partial *r*: the size of the effect in terms of the partial correlation coefficient; ncp: the non-centrality parameter for the effect.
